# Barriers to the Implementation of the Health and Rehabilitation Articles of the United Nations Convention on the Rights of Persons with Disabilities in South Africa

**DOI:** 10.15171/ijhpm.2016.117

**Published:** 2016-08-28

**Authors:** Meghan Hussey, Malcolm MacLachlan, Gubela Mji

**Affiliations:** ^1^Centre for Global Health, Trinity College Dublin, Dublin 2, Ireland.; ^2^School of Psychology, Trinity College Dublin, Dublin 2, Ireland.; ^3^Centre for Rehabilitation Studies, Department of Interdisciplinary Health Sciences, School of Medicine and Health Sciences, Stellenbosch University, Cape Town, South Africa.

**Keywords:** Disability, Convention on the Rights of Persons with Disabilit, South Africa, Implementation, Barriers

## Abstract

**Background:** The United Nations (UN) Convention on the Rights of Persons with Disabilities (CRPD) is a milestone in the recognition of the human rights of persons with disabilities, including the right to health and rehabilitation. South Africa has signed and ratified the CRPD but still has a long way to go in reforming policies and systems in order to be in compliance with the convention. This paper seeks to fill a gap in the literature by exploring what the barriers to the implementation of the health and rehabilitation articles of the CRPD are, as identified by representatives of the disability community.

**Methods:** This investigation used a qualitative, exploratory methodology. 10 semi-structured interviews of a purposive sample of representatives of disabled persons organizations (DPOs), non-governmental organizations (NGOs), and service providers in South Africa were conducted. Participants were drawn from urban, peri-urban, and rural settings in order to reflect diverse perspectives within South Africa. Data was analysed using a multi-stage coding process to establish the main categories and relationships between them.

**Results:** Six main categories of barriers to the implementation of the health and rehabilitation articles of the CRPD were identified. Attitude barriers including stigma and negative assumptions about persons with disabilities were seen as an underlying cause and influence on all of the other categories; which included political, financial, health systems, physical, and communication barriers.

**Conclusion:** The findings of this study have important implications for strategies and actions to implement the CRPD. Given the centrality of attitudinal barriers, greater sensitization around the area of disability is needed. Furthermore, disability should be better integrated and mainstreamed into more general initiatives to develop the health system and improve the lives of persons living in poverty in South Africa.

## Background

It is estimated that over 1 billion people worldwide have a disability, the majority of whom live in low- or middle-income countries (LMICs).^1^ People with disabilities are three times more likely to report being denied medical care and are four times more likely to report receiving sub-standard care.^1^ Despite these statistics, persons with disabilities remain one of the most marginalized and under-studied populations in global health.^1^

The 2007 United Nations (UN) Convention on the Rights of Persons with Disabilities (CRPD) is a milestone international treaty to protect and promote the full and equal rights of persons with disabilities.^2^ Although the rights of persons with disabilities were implicitly included under previous UN human rights agreements, such as the Universal Declaration of Human Rights and the UN Convention on the Rights of the Child, the CRPD is the first document that enshrines the rights of persons with disabilities with specific concerns that must be protected.^3^

Despite the ratification of the CRPD by 166 countries, much work still needs to be done to bring countries’ domestic laws and systems into accordance with the convention.^4^ There is a stark disconnect between the vision laid forth in the CRPD and the reality experienced by the majority of people with disabilities in the world. What should be happening according to the existing policies is too often not being transferred in practice. This research sought to investigate why this is so by exploring barriers to the implementation of the health and rehabilitation articles of the CRPD in South Africa.

South Africa has signed and ratified both the CRPD and the optional protocol in 2008.^4^ However, the country still has a long way to go in order to be in full compliance.^5^ Approximately 2.8 million South Africans over the age five are classified as disabled.^6^ South Africans with disabilities face significant challenges in the areas of health and rehabilitation. People with disabilities in South Africa are far more likely to self-report having poor health than people without disabilities.^7^ It has been estimated that from 2002-2008 children with disabilities in South Africa were 2.5 times more likely to be ill or injured than their non-disabled counterparts.^8^ Small-scale surveys conducted in South Africa at the township and district level found only approximately a quarter to a half of children with disabilities were receiving rehabilitation services.^8^ One study in Johannesburg showed that significantly fewer people with disabilities reported being covered by medical aid, which would render them less likely to be able to pay for healthcare despite the possibility they would have higher costs.^7^

The lack of data about persons with disabilities specifically has been a major impediment for government policy and planning, as well as for the inclusion of disability in the larger global development agenda.^9^ In South Africa, it has been argued that research on disability has not been conducted in a way that can contribute to the development of concrete solutions or effective advocacy.^10,11^ Specifically, the lack of qualitative data from South Africa has frustrated efforts to identify where the difficulties lie in successfully implementing good disability policies.^5^ Globally there is a need for research to be specially targeted on the CRPD implementation process.

This paper seeks to fill this gap in the literature by exploring what the barriers to the implementation of the health and rehabilitation articles of the CRPD are, as identified by representatives of the disability community.

## Methods

A qualitative, exploratory approach was taken to determine community-identified barriers to implementation of the health and rehabilitation articles of the CRPD. The main source of data was 10 qualitative interviews conducted by the first author in South Africa. Interviews followed a semi-structured interview schedule; allowing the researcher to both have a set of questions as a guide but maintain flexibility to follow up and probe further information that emerged.^12,13^ Questions were initially left broad (see Box 1) to allow participants to introduce topics that may not have been considered by the researcher. Following giving consent, interviews lasted between 33 and 75 minutes, were audio recorded and notes were taken as both a backup and to document facial expressions and body language.

**Box 1. Interview Guide**

Article 25 of the Convention on the Rights of Persons with Disabilities (CRPD) says: “States Parties recognize that persons with disabilities have the right to the enjoyment of the highest attainable standard of health without discrimination on the basis of disability. States Parties shall take all appropriate measures to ensure access for persons with disabilities to health services that are gender-sensitive, including health-related rehabilitation.”

1. What do you think are the major barriers to accessing healthcare for persons with disabilities in South Africa?

I. What are the physical barriers you have seen for persons with disabilities in accessing healthcare?
II. What are the communication or attitude barriers to health for persons with disabilities?
III. Are you aware of reports of persons with disabilities being discriminated against in healthcare?

2. What are the other barriers in the lives of persons with disabilities that you have observed have an impact on their health? Article 26 of the CRPD says: “States Parties shall take effective and appropriate measures, including through peer support, to enable persons with disabilities to attain and maintain maximum independence, full physical, mental, social and vocational ability, and full inclusion and participation in all aspects of life. To that end, States Parties shall organize, strengthen and extend comprehensive habilitation and rehabilitation services and programs, particularly in the areas of health, employment, education, and social services…”

1. What are the barriers for persons with disabilities to accessing existing rehabilitation programs?
2. What do you feel are the challenges in extending or expanding comprehensive rehabilitation services for persons with disabilities?
3. Why do you think these barriers to health and rehabilitation exist for persons with disabilities?
4. What do you think the government should do to eliminate these barriers?

Field notes were taken during data collection and used to supplement the interview findings. Field notes included passive observations at organization’s facilities or the setting of the interviews. The websites of organizations from which interviewees were drawn, and grey literature about South Africa’s health and rehabilitation system, were also reviewed.

### Participants

This study used a non-probability purposive sample of 10 representatives of disabled persons organizations (DPOs), non-governmental organizations (NGOs), and service providers that work with and on behalf of persons with disabilities, who both know the concerns of the communities and have put themselves in a position to advocate on their behalf. Five of the respondents were persons with disabilities themselves, while two were parents of children with disabilities, and three were professionals including one social worker, one occupational therapist, and one rehabilitation coordinator. A list of 10 purposefully selected organizations was drawn up to get a diverse picture of the concerns within various subgroups within the disability community, including physical disabilities, sensory disabilities (sight or hearing), and intellectual/developmental disabilities. Inclusion and exclusion criteria are represented in Table. These organizations were nominated on the basis of expert advice from the Chair of the African Network for Evidence to Action on Disability (AfriNEAD), who has worked in this area for over 20 years.

**Table:** Inclusion and Exclusion Criteria

**Inclusion Criteria**	**Exclusion Criteria**
• Over the age of 18 • Has the ability to give informed consent • A representative of a DPO or organization that represents or works on behalf of persons with disabilities in South Africa • Able to meet for an in-person interview in one of the three study settings	• Children under the age of 18 • Presents with an intellectual or cognitive impairment that impedes ability to consent • Declined to participate in the study • A person with a disability served by an organization living in poverty (making them a vulnerable group member)

Abbreviation: DPO, disabled persons organization.

### Setting

**Figure 1:**
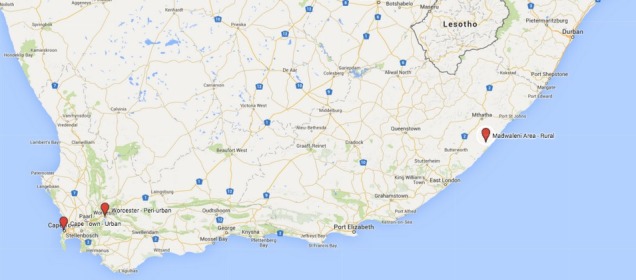
Map of Research Settings (Produced by author using Google Maps).

Participants were drawn from three different types of settings in South Africa: urban, peri-urban, and rural (Figure 1). The decision to sample from across the rural-urban spectrum was made in consultation with advisors from Stellenbosch University, Stellenbosch, South Africa; as they felt that it was important to account for disparities between different South African communities and to minimize the potential bias of convenience sampling in one location.

The first stage of participant recruitment took place in urban areas around the Cape Town metro area, Western Cape province, where five interviews were conducted.

The second stage of sampling took place in the peri-urban town of Worcester in Western Cape province, approximately 1.5 hours drive outside of Cape Town. A total of three participants were interviewed during this stage of research.

The rural setting included a series of villages in the catchment area of the Madwaleni Hospital, in a remote area of the Eastern Cape province. This collection of villages was over 4 hours drive from the city of East London and the landscape was characterized by traditional round houses and rough gravel roads winding through the steep hills. Two interviews were carried out during the rural stage of data collection.

### Data Analysis

Data collection, transcription and analysis took place concurrently in order to generate new ideas, allowing interview questions to evolve as additional data was collected.^14^ Interview transcripts were analyzed using a multi-stage coding process. Interview transcripts were labeled using open coding and the list of codes was recorded. The codes were emergent, coming directly from field data rather than preconceived ideas of the researcher.^15^ Codes that shared similar characteristics were then grouped into categories.

Following open coding, axial coding was used to determine connections between the categories. Axial coding using the causal-conditional matrix can draw out connections of micro and macro conditions and consequences, thus, making it particularly fitting for research questions focused on barriers and policy implementation.^16^ This approach first looks at the phenomena of interest, then possible causal conditions and contexts. Finally, selective coding was used to determine the final themes. All concepts that emerged were considered, with those that came up repeatedly being considered most relevant and grouped into themes.^14^

## Results and Discussion

**Figure 2:**
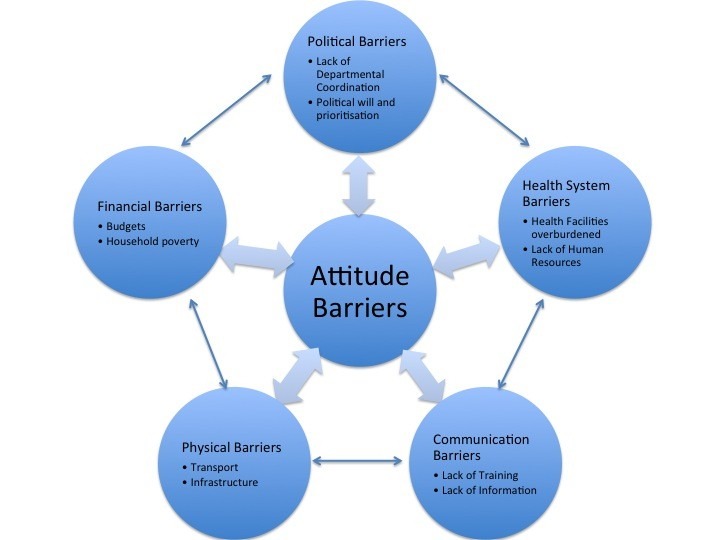
Categories and Relationships of Barriers.

After the final data analysis, six major categories of barriers were identified: attitudinal barriers, political barriers, financial barriers, health system barriers, physical barriers, and communication barriers. It is also important to understand how these barriers interact with and influence one another in ways that impede the implementation of the right to health and rehabilitation. The relationships identified between these are represented in Figure 2. These barriers influenced one another through a series of reciprocal connections. In some cases, the connections between categories were mediated by another category. For example, financial barriers influenced health system barriers, but this connection was associated with political barriers regarding increased funding. All of the categories on the outer circle were influenced by the category of attitudinal barriers, which emerged as being central.

### Attitude Barriers

The first category of barrier explored was attitudinal barriers, which participants identified as central and linked to each of the other barriers. While barrier removal is an undertaking that involves technical and financial solutions, it is also a question of policies and the attitudes that influence these policies.^17^ Respondents talked at length about the need for a mindset change around disability in South Africa, which supports the South African Human Rights Commission statement: ‘prejudice remains the greatest disability,’ and observations by previous research.^18,19^ All participants cited stigma and negative perceptions towards persons with disabilities in South African society as being the major underlying barrier to health and rehabilitation. A challenge to the practical implementation of the CRPD, therefore, lies in overcoming barriers of attitude, which may also be associated with culturally-based beliefs.^20^

Some of these attitudinal barriers indicated by participant clearly illustrated the importance of cultural interpretations of disability.

*“It’s a cultural thing to hide your child with a disability. You do not want people to see this because in some cultures it’s seen as, you know, ‘the gods, the forefathers are frowning upon the family’ and, you know, cursing you”* (Female, Urban).

*“They are hidden. They didn’t want to be seen, these people. They don’t want to be seen because of the stigma, of the stigma” (Male, Rural)*.

Respondents spoke about the attitudes that perpetuate social exclusion of persons with disabilities. This caused a tendency to forget that while persons with disabilities may have health needs due to their specific physical or mental impairments, they also need to access general health services. For example, persons with disabilities can also contract HIV/AIDS, yet have often been excluded from HIV/AIDs prevention and treatment programs.^21^ One participant described this conception of persons with disabilities as “other”:

*“When I say ‘us and them,’ there’s still that thing that ‘we are able bodied’ and forgetting that what affects us as abled bodied people may also affect people with disabilities. They’ve got everything; they’ve got needs like us. It’s just that there’s this element that makes them different from others. There’s this extra need by them”* (Female, Rural).

Attitudes were identified as either a direct cause or influencing factor for all of the other types of barriers that were identified.

### Political Barriers

#### Lack of Coordination

The first element within political barriers was confusion of exactly which department within the government is responsible for policy concerning persons with disabilities. The situation is well-articulated by the following quote:

*“The last cabinet used to have a specific department for women, children and people with disabilities, in itself quite a ludicrous combination. You know? Women, children, and people with disabilities. Oh my gosh. How did that all end up in one department? Just like the vulnerable sectors of the world, let’s just throw them into one little department, let one department deal with the entire mess. That department’s now been scrapped and all of those things have been brought into the National Department of Social Development, which in a way is a good thing in that ‘the buck has to stop’ there. The bad thing is that there is no longer any formal recognition of the needs of women, children and persons with disabilities. There is lack of commitment and the lack of priority of those issues”* (Female, Urban).

The effects of this instability were described on both a macro level; when a new cabinet comes into power and there is associated reorganizing of national government departments; but also on the local level, where it may come down to the instability of having individual politicians driving change.

*“I think at a political level the tables get turned depending on which political party is in power and sometimes you have a switch to giving you this only or that only and then you sort of throw the baby out with the bathwater instead of building on what you have”* (Female, Urban).

*“I also find that often it’s driven by individuals so if there’s a change in leadership, someone gets a promotion, moves to a different department then all the momentum that they developed will just stop”* (Male, Urban).

“Line functions” and “silos,” where different government departments operate in isolation of one another, were often terms that were used to describe government departments, including the departments of health, education, and social development. While mention of inter-sectoral coordination is mentioned in the South African Department of Social Development’s Disability Policy (Department of Social Development), community members found that partnerships for collaborative and holistic service delivery had not been established effectively.

*“So they work in their silos of education, health, social development, and very rarely do they actually work horizontally across those silos. So it makes it easy to shift it into a different department so that nobody ever takes responsibility for it”* (Female, Urban).

The lack of coordination between different government departments is illustrated by the following example:

*“I can’t tell you how many times we [noted in] meetings [that] Cape Town City Council wanted a disability policy…That department, whichever department it was, which was trying to pull this all together was not communicating to the rest of the other council departments because you have a policy that says that all buildings should be accessible or transport should be accessible. So you go to transport department and say: “do you know about the inclusion bill of person with disabilities?” and they go “what?” So even within the city council, which wanted this wonderful piece of legislation, they weren’t informing departments. That’s the same with government because you’ve got a hospital, ok? They’ve got money to treat people. They ain’t got money for transport. You know? So what’s the point of trying to treat people if people can’t get there? Then you have to have two departments working together and they’re not [hits table] working [hits table] together!”* (Female, Urban).

#### Attitude of Political Leaders

An underlying driver of these political barriers was the perceived attitudes of the government towards persons with disabilities. One of these was ignorance:

*“As far as the constitution goes, the South African constitution has all the right words. We have all the words in place but there is still an attitude, which makes me very mad sometimes. I think it’s ignorance, the lack of education around how we deal with a situation”* (Female, Urban).

In rural areas, local leaders such as village chiefs were identified as another key group that needed to be empowered to use and implement the CRPD in their communities.

*“But I think another barrier is lack of insight, it’s lack of insight with the leaders”* (Female, Rural).

Other researchers have echoed this finding that politicians and leaders have inadequate understanding of disability, particularly from a human rights perspective.^22^ This is related to how political responsibility for the implementation of disability policy is taken by government departments. For example, research on wheelchair provision in South Africa found that lack of knowledge among provincial managers about the practical challenges was a leading cause of the lack of implementation of the assistive device mandates of the CRPD as well as the South African Department of Health Guidelines.^23^

Another attitude barrier was the value and seriousness with which persons with disabilities were handled.

*“These people are not taken seriously, even by the government. Even the government doesn’t take them seriously”* (Male, Rural).

Inability of the government to seriously consult and involve the disability community and leaders at more grassroots level was also noted. Participants expressed a desire for more interaction between government officials and the disability sector.

I: What do you think the government should do to eliminate these barriers to implement the convention?

*P: Change their attitude. I think it’s attitude and government should consult and hear…They need to speak to the people here, the people that’s in need of the service. And if they can involve the people that’s in need of the services and the people on ground level, the individuals, consultative forums…Take the forum to the people* (Female, Peri-Urban).

*“You see the other thing, speaking with this minister that we’ve got, it’s like you’re there to represent people with disabilities and you’ve never met with the sector. What’s going on? Your first job as minister is to meet with the sector and find out what we need. But no it’s we’ve got to sit through all of these endless, endless dark meetings and then you kind of wonder ‘well she ain’t going to read that. Some lacky [a footman or servant] in the office is going to make a summary of it.’”* (Female, Urban).

Finally, this attitude and lack of understanding resulted in disability being “stand-alone,” rather than mainstreamed into health policy.

*“I would say because of society’s understanding of disability, because disability is not fully incorporated into our healthcare services. It’s sometimes a ‘stand alone,’ it should actually be part of all healthcare services and you know? And interventions. It shouldn’t be seen as a stand-alone”* (Female, Urban).

These findings identify attitude of disability as an “afterthought” or “add on” in policy and reinforce the call of Schneider et al^24^ for the needs of persons with disabilities to be mainstreamed into policy documents that set healthcare priorities.

#### Financial Barriers

##### Budgets

The first subcategory under financial barriers was budgets, which was linked to political barriers. Politicians make decisions about where resources are allocated, however, they are constrained by the limited size of the financial pie. In this way, political barriers serve as a mediator for the connection between financial barriers and health system barriers. Respondents identified the connection between political barriers and financial barriers.

*“In South Africa we have the best constitution on paper with really meager resources made available to implement the fancy constitution that we have on paper…”* (Female, Urban).

*“Finance. Funding, finance, and funding and finance. I think sometimes about the way money is spent in this country that could be better utilized just making a difference”* (Female, Urban).

“*There is no budget for the disabled. No clear budget for* t*hese people. There is no clear budget. They say ‘we are going to do this and that and that.’ They just say! Nothing is done for them”* (Male, Rural).

Political barriers influence financial barriers because political leaders set the priorities and make value judgments on how money should be spent. When asked what do you feel are the challenges around extending or expanding comprehensive rehabilitation services for persons with disabilities? One participant responded:

*“I think funding. State priority, yea. The Department of Health’s priorities. It depends, you know, in their opinion what are the key areas where they need to intervene. And you know at the moment it is in the HIV/AIDS field so I think there is more of an emphasis to provide services on that side. So for the rehabilitation it’s not that it’s not important, not that much funding goes to rehabilitation the way it goes to HIV”* (Female, Urban).

At the same time, respondents realized that political barriers were also created by the real limitations on the budget and financial resources of the government.

*“So even though the sector is coming to the government the government is not facing it because the government is always going to say: ‘well we can’t do that because we haven’t got the money’”* (Female, Urban).

*“So yea we could definitely use a massive increase in budget but I’m sure every government department will say the same thing”* (Male, Urban).

Finally, attitude barriers were seen as another underlying factor, as participants felt that the government did not see persons with disabilities as an economically smart investment.

*“We speak. We pay lip-service to the idea of inclusion and meeting the needs of people with disabilities, but the reality is that money [economic growth] is seen as a priority on which to spend money”* (Female, Urban).

Budgeting involves value judgments and trade-offs. Therefore, if persons with disabilities are not valued, if politicians are ignorant about disability issues, or do not consider their needs a worthwhile investment, this will be reflected in the budget.^25,26^

#### Poverty

The second sub-category was poverty at the household and individual levels. The positive correlation and mutually reinforcing relationship between disability and poverty is well-established in the literature.^27-30^ Poverty and income inequality is a major social determinant of access to healthcare and eventual health outcomes.^31-34^

Attitudinal barriers heavily influenced poverty. Social exclusion of persons with disabilities was described as a major contributor to the poverty experienced by people with disabilities in South Africa. Participants mentioned persons with disabilities being hidden and denied not only health services, but also education and employment. This was more common among families living in poverty, who are marginalized overall. The following story regarding a participant’s experience in an impoverished informal settlement community is an example:

*“Two weeks ago I went to work and there was a girl in a wheelchair and she was lying on the side and she was about 23 years old and everyone is amazed she survived. And she was found 6 months ago in her parent’s shack. They had built a shelf for her above the parents’ bed and they used to bathe her, feed her, strap her in there, and then go out and work for the whole day. They had no money for wheelchairs. They didn’t have the education to take her to a [rehabilitation] centre or anything like that, they literally didn’t know anything 22 years ago or whenever she was diagnosed. Where was her help?”* (Female, Urban).

The majority of participants brought up financial assistance from the government. Means-tested disability grants are available to persons with disabilities over age 18 (DSD 2015). A Care Dependency Grant is available for parents of children with disabilities. Currently the amount is a maximum of 1410 rand per month. The grants were often reported as being a family’s only source of income due to high unemployment and poverty within South Africa,^35^ as detailed in the following quotes:

*“Now government has made access to the grant to the disability grant, which will actually enable the mother to pay for transport to be able to take the child for services. But unfortunately most [people with] disabilities come from poverty-stricken areas where actually the grant will be used to feed the rest of the family. You see? It will end up with the mother not being able possibly to bring the child to the next follow-up clinic for management [the child’s therapy] because all the money has been used to feed the family”* (Female, Rural).

*“I would hazard a guess that probably 80% of kids with disabilities are being raised by single mothers, so you then have that whole vicious circle and cycle of mom can’t go to work because the child needs specific care. There is no money. There is a disability grant and then the disability grant feeds the whole family, as opposed to providing the needs of the child. So we have that abuse of funds that are made available as well”* (Female, Urban).

The use of the grants collectively was identified by participants in this study as a barrier because this prevented the money from going to meet the health or rehabilitation needs of the person with a disability. Previous research undertaken in the rural study site found that families in poverty were making decisions constrained by the cultural context and burden of poverty rooted in historical social suffering.^36,37^ The South African Disability Grant program remains one of the few means of social assistance for families living in poverty.^38^

The poverty experienced by many people with disabilities and their families both influenced and was influenced by physical factors such as transport. This was a vicious cycle because without transport to health facilities or social security offices, persons with disabilities were unable to apply for financial assistance that would then help them pay for further health or rehabilitation services.

“*And even to find their birth certificate, it’s so difficult because they sometimes give birth there in the rural area because there’s no hospital, it’s far and the clinic is far and it’s only attended by these old mamas, these old mamas. Even after she gave birth it’s difficult for them to get the child to the clinic or to the hospital. Because the mother doesn’t have money…”* (Male, Rural).

This has come up in previous research both in South Africa and in other LMICs contexts.^37^

An understanding of the collective nature of traditional South African culture is important when trying to unpack the issues of the financial barriers around household poverty. A key South African concept to understand is “ubuntu,” in which people are defined through their relationships and community.^39,40^ In the case of disability, where there is often a large stigma attached, this means that it is not only a “disabled person” but also a network of disability-related relationships, a “disabled family”^41^ that needs to be considered; and the economic consequences of a person with disability on that family network. A person with a disability is, therefore, inextricably linked to their family, who also experience marginalization.

The implications of this for the implementation of the CRPD are considerable. It demonstrates that the rights of persons with disabilities are tied to the social and economic rights of their families and their communities. Just as thinking and working in silos has proven to be a political barrier for the implementation of the CRPD, the same is true when financial assistance measures to improve the lives of persons with disabilities attempt to divorce the needs of the wider family living in poverty. When combined with negative attitudes towards disability, it may be decided by some that spending limited resources on the health of a family member with a disability is a poor investment and priority should be put on other needs.^26,42^

The implementation of the CRPD is, thus, incumbent on rights-based approaches to development and global health. Some authors have suggested that the disability grant or care dependency grant be repackaged in a way that provides specifically for services that would enhance the functioning of the person with the disability such as assistive devices, rehabilitation, or skills training, thus, allowing for persons with disabilities to contribute to the collective needs of the family.^43,44^ This was in keeping with the suggestion made by several participants who worried that the grant created a “dependent society” and the need to move towards an attitude of “seeing ability over disability.”

### Health System Barriers

#### Overburdened Facilities

Participants cited long queues and waiting times in overburdened health and rehabilitation facilities. Often these are due to the high demand for health services in the state facilities overall, rather than something specific to disability. However, the long waiting times or overload place an undue burden on patients with disabilities seeking care. For certain types of disabilities, the overload can lead to inadequate care that is not only detrimental to the individual’s health, but also to the overall health system.

*“Because of the pressure on beds the rehabilitation period has been reduced. So people are not getting the rehabilitation they require. They’re being discharged into the community on a much lower level than they should be…And then because of that people don’t know; it’s a vicious circle. People don’t know how to look after themselves properly; they still have to come to terms with what happened to them. I know they develop secondary issues, which then puts a drain on the healthcare system”* (Male, Urban).

Furthermore, the overload had a disproportionate impact on persons with disabilities requiring assistive devices. Long waiting times of up to two years for state-provided wheelchairs were described by several participants. These overloads and waiting lists were linked to financial barriers in the form of budgets, as filtered through the priorities of the political system.

“*When we look at, for example, the process of getting a new wheelchair. If you’re in the government queue you have to go to your local clinic. They know nothing about your wheelchair. They are supposed to refer you to the rehab center and at the rehab center the waiting list is probably 2 years. Then when you get there, there’s only so much money, so you won’t get the right wheelchair. You’ll get a new wheelchair but it won’t be the right wheelchair”* (Female, Urban).

This participant went on to elaborate as to why this was the case, which came down to an attitude barrier:

*“Well it’s because it’s a kind of ‘one size fits all’ attitude and that’s not right. You need to provide assistive devices that are going to make the person’s life easier regardless of money”* (Female, Urban).

Analysis of the South African health system has found that delivery and financing of care is highly inequitable and skewed to favor the rich who can afford private medical care.^45^ The long queues and waiting times described are a consequence of the fact that the demand for health and rehabilitation services far exceeds the ability of the government to deliver. This is a persistent challenge to the realization of universal health coverage in South Africa overall.^46^ However, this limitation particularly affects persons with disabilities.

#### Lack of Human Resources

Lack of health professionals trained in specialized skills relating to health-related rehabilitation was identified as a large barrier. While participants from Cape Town lauded professionals working at facilities such as the Western Cape Rehabilitation Centre, no such professionals were available in the remote area of the Eastern Cape.

*“So lack of personnel in rehabilitation is a key we need now because if we don’t have this kind of, we need to empower other people, because we have other health professionals”* (Female, Rural).

One participant described the lack of specialized health extension workers for persons with disabilities in rural communities, citing the fact that they were available for other conditions.

*“You will find people called ‘care workers’ for HIV only. There are none for persons with disabilities. You cannot find them. They only visit people who are the HIV+; they do not work with people with disabilities. I never…I never find someone who says ‘I am the care worker of the disability people.’ No. No person is working with people with disabilities. Is there any care worker for that? No, no, I never saw them”* (Male, Rural).

According to the baseline CRPD implementation report from South Africa,^5^ such a disability module does exist in the training for nurses, however, participants said that they were not aware of it. However, this type of training is explicitly mentioned in Article 25(D) and Article 26.2 of the CRPD and reforming training curricula could constitute of low cost intervention to implement.

However, it was felt that the presence of attitude barriers meant not all health personnel would be open to the disability training that participants felt was necessary.

*“It’s still a difficult process and it’s still something that people are still afraid of persons with disabilities. They just, and I do have to say not all persons are comfortable with even learning or becoming sensitized with persons with disabilities”* (Female, Peri-Urban).

Community-based rehabilitation (CBR) has been promoted by the World Health Organization (WHO) to provide comprehensive rehabilitation services to people in low-resource settings.^47^ Often the CBR workers in these programs are persons with disabilities themselves, making them particularly effective for overcoming communication and attitude barriers. This also fulfills the suggestion of Article 26 of the CRPD that peer support be included within rehabilitation services. Some participants did cite examples of CBR, but these were specific examples being implemented by organizations in certain provinces, rather than as part of a larger overall national program:

*“Community-based rehabilitation. Look, as far as I know we’ve got a program out in Mpumalanga province, so that is very available for persons with disabilities. Also, we’ve got a program out in Kwa-Zulu Natal, a community-based rehabilitation program. The Department of Health both in Mpumalanga and in Kwa-Zulu Natal are both very supportive of our organization in the implementation of these programs. The other provinces we’re still trying to convince, especially the provincial departments of health, that they need to implement these programs. Because what we do is use persons with disabilities themselves to actually implement the program because we found that peer support works much better”* (Female, Urban).

However, CBR and other peer-support systems faced difficulties in financial sustainability.

*“Peer supporters are very effective. I do think though that there’s a question whether they’re sustainable or not. People get trained and because there’s no or very little enumeration they always have to look out for something else”* (Male, Urban).

While this approach has been piloted in rural South Africa since the 1990s, barriers to scale up exist.^48,49^ First, it was incumbent upon the provincial or district government bodies to be onboard with such a project, as it was not a nationally rolled out strategy. Second was the issue of financial compensation for peer-supporters and CBR workers, is a constant challenge to the model.^50^ Third was that CBR is an inter-sectoral approach and requires cooperation between different departments, lest it fall back into the medical model of disability rather than a holistic approach to rehabilitation.

#### Physical Barriers

##### Transportation

Lack of accessible transportation (for instance, buses) for persons with disabilities to access health or rehabilitation services and the actual movement of people to the places where health and rehabilitation services are provided proved to be a major barrier.

*“Well transport’s number one, and that’s the biggest barrier that we have…Often people aren’t able to make the trip themselves so someone will have to stay out of work to take them. Sometimes children stay out of school to help their, accompany their parents, and parents don’t want to do that. They’d rather their kids go to school so they just don’t go to hospital”* (Male, Urban).

Attitude barriers also influenced transportation. Whereas some non-disabled people without cars could make use of taxis or public transportation, lack of sensitization or poor attitudes made these inaccessible or unsafe for persons with disabilities.

*“And so it’s particularly bad for mothers with children in wheelchairs because taxis won’t pick up the wheelchairs. So I’ve seen mothers have to carry children and young adults with severe disability on their back to get them to the hospitals”* (Female, Urban).

*“Often our public transport is definitely not sensitized towards persons with impairments. And then with regards to safety some of our residents, because of the fact that they can’t see well because they’ve got low vision, we’ve we had one of our residents that fell in front of the taxi. Fortunately he wasn’t hurt badly, but he hit the tar quite hard because he couldn’t see the step and no one was there to assist him”* (Female, Peri-Urban).

The disparity in transport available in urban and rural areas was recorded in field notes. Transportation systems that were disability inclusive, such as Dial-A-Ride and MyCiti bus, were found in the city centre of Cape Town but were limited in capacity. These were not available in the rural areas, where the need was arguably greater due to increased distance between health facilities and poor roads.^23,51^ Shared mini-bus taxis were the only public transport available and were not accessible for someone with a disability unaccompanied and definitely not for someone with a physical disability.

*“They are coming very far, to up here, and this person is disabled or the transport to transport this person to come here they have to hire a car, it’s very expensive to hire a car to get to here”* (Male, Rural).

This reinforces the finding by Maart and Jelsma,^52^ who found that 72% of people with disabilities in a deprived area of Cape Town said transport was the main problem with accessing services. Similarly, Saloojee et a1^53^ found that in a peri-urban area of South Africa one return trip for rehabilitation therapy at a hospital 30 km away consumed as much as 5% of a family’s monthly income.

##### Infrastructure

Physical infrastructure of health facilities was also a barrier. Many health facilities were described as being old and out of line with universal design and accessibility. Newer buildings also did not always meet international accessibility standards. This was interpreted as a form of discrimination against persons with disabilities in the health service.

*“I would say discrimination is there, because nowadays I cannot understand how you put any structure in place without accommodating accessibility in case of people with disability. You get access to the building if you’ve got a lift. There must be an auditory kind of communication for those who cannot see or who are blind. I mean there are modern buildings where you go into it and you go ‘ah ah,’ this is too modern for it not to have certain, you know, features that would cater for people with disabilities. So for me that’s* discrimination” (Female, Rural).

One example that came up among several participants was the lack of lifts and building requirements for wheelchair users.

*“For wheelchair users, we need more space. We need ramps, hoists, etc. So it’s not just for operations, but also infrastructure for facilities”* (Male, Urban).

Another example of physical infrastructure adaptations for persons with disabilities that were missing included signage. For example, tactile or color coded markings or navigation systems would make health facilities accessible for persons with visual impairments.

*“When they reach the hospital there’s no signage, no method of indicating where they need to be if they can’t see the visual markers [signs]. That makes it quite difficult. We in South Africa, we’re quite a bit behind other countries with tactile markings. They are something that you find quite often in other countries but in South Africa we don’t have that yet”* (Female, Peri-Urban).

##### Communication Barriers

###### Communication With Health Professionals

The first sub-category was difficulty in communication between patients and health practitioners. Another quantitative study done in South Africa, found that 48.3% of people reported communication or language as a barrier to accessing services.^52^ The results of this study expand on that knowledge by pointing out that these communication barriers are often linked to attitudes or lack of training. Attitudinal barriers in the form of negative or incorrect assumptions about persons with disabilities were often explicitly linked to communicational barriers. A general lack of awareness about how to interact with persons with disabilities or incorrect assumptions made the provision of healthcare inaccessible or inadequate for persons with disabilities. This causal link is a salient feature of the following quotes:

*“There’s once again the assumption that relates to the attitude that’s often the mere function of seeing someone in a wheelchair. The assumption is that the person cannot speak or cannot speak for himself and is cognitively impaired as well. So, it’s that whole bundle of assumption and prejudice on people with disabilities. That creates a break in communication”* (Female, Urban).

*“I think a lot of time and effort is wasted because there’s this assumption that because I can’t speak I’ve got nothing to say”* (Female, Urban).

This is regularly cited as a barrier not only to the access of healthcare, but the quality of healthcare, which is cited in Article 25a and d of the CRPD.^1^

Many respondents described health practitioners as falling into traps of speaking to the ‘carer’ or accompanying person, rather than to the person with a disability presenting for care. One respondent gave an example:

*“For example, if I was to go to medical facility on my own and my speech wasn’t good, they wouldn’t automatically assume, ‘Well she’s on her own she must know what she’s talking about.’ Or if I presented with someone, they would tend to talk to the other person rather than to me. I had surgery on my shoulder once and my husband was there and they started talking to him instead of to me”* (Female, Urban).

This communication barrier had further impacts on the protection of the CRPD enshrined rights of patients with disabilities to autonomy and confidentiality.

*“Then again even if they are really close to the staff member who takes them it’s still a question of personal space and privacy and all of that. You have to be able to share all of your most private thoughts, medical experiences with a third party”* (Female, Peri-Urban).

Lack of sign language interpreters was a major barrier to accessing equitable health services for Deaf persons. One example of just how lack of communication impedes the realization of the right to health for persons with disabilities was described:

*“There’s a good example I can give you in terms of healthcare services. One of our Deaf persons was admitted for Tuberculosis and HIV/AIDs. She got treatment and during her the time at the HIV/AIDS the nurses just came and they just give it [the medication] to her and she drank but no one explained. So obviously the patient would take the medication. Now the person is discharged from the hospital and they give the packet of medication with no explanation. For the four weeks that you’ve been in the hospital we have given the mediation so you should know what to do. That patient never drank the medication so she died. So what is that? It’s because of the lack of knowledge from the medical part but it’s the lack of communication. So communication is huge”* (Female, Peri-Urban).

Barriers to communication within the health system for Deaf persons are documented in South Africa, where a shortage of interpreters makes it particularly difficult for patients to communicate with providers or access care.^19,53,55^ Civil society organizations have argued that the CRPD compels states to provide sign language interpreters, but there has still not been a good model of implementing this in a sustainable way within the health system.

###### Lack of Information

The second sub-category was barriers in communicating what is available in terms of services. In some cases, participants described services and programs being available but without sufficient communication links to make potential users of the services aware.

*“I think there’s also a lack of knowledge of what is out there, what facilities are there, in our case there are quite good facilities but people aren’t aware of them or don’t know how to access them or know where to go”* (Male, Urban).

This was linked to health system barriers. The breakdown in communication would be improved by linking the formal health system and NGOs that provide support and/or services. Previous researchers have noted that lack of awareness about health and rehabilitation services is a key reason that they are under-utilized by parents of children with disabilities.^53^ This has severe consequences for the child with a disability over their life course. One participant described the need for information about disability and available services:

*“Information. Availability of information from different organizations; it doesn’t have to be a public service. There are so many NGOs or people with expertise that want to assist. All health facilities must be provided with contact details of the organizations and people with expertise who would assist those people [with disabilities] to access services that would assist their families, their children, parents, whatever, who suffer from whatever kind of disability”* (Female, Rural).

Another participant brought up a platform for cooperation between local government and the disability sector as an effective strategy to address this:

*“So in Worcester we have quite an active multi-sectoral action team, it functions like a forum, but with more action to it than just getting together and talking about services. So we talk more about how to reach out to different departments, how to make persons aware about different services within the community”* (Female, Peri-Urban).

##### Limitations

This research was subject to a number of limitations that must be considered in the appraisal of the results and discussion. Fieldwork had to be carried out under a constrained time period of 5 weeks. The researcher, therefore, could not spend a significant amount of time doing community entry in the field, which would have afforded a better understanding of context. Interviews were all carried out in English. South Africa is a linguistically diverse country, with 10 official languages. As such, only having English-speaking individuals possibly influenced and limited the perspectives represented. Finally, this study’s research question, ethical approval, and access was limited to members of the disability community and civil society. Other key stakeholders who may be able to shed light on the barriers to the implementation of the CRPD, such as government officials and persons with disabilities who are unaffiliated with any formal organization, were not sought.

### Conclusion

The purpose for undertaking this research was to determine where the barriers to implementation of the health and rehabilitation articles of the CRPD lie in South Africa. The results of the study indicate that the barriers to the implementation of the health and rehabilitation can be found in the realms of politics, finances, the health system, the physical environment, and communication. Most importantly, persistent underlying attitudinal barriers within South African society that stigmatize and exclude persons with disabilities influenced all of these barriers.

As Figure 2 illustrates, the interconnected nature of the emergent categories means that a systems approach is needed to addressing barriers to the effective implementation of the CRPD, regarding health and rehabilitation. That is intervention at one level may be ineffective unless corresponding facilitating actions are taken at other levels. Consequently coordinated action must be taken at all levels, from individual and community behavior, to how services are organized and delivered, to how policy addresses vulnerable or marginalized groups, through culturally and contextually informed and effective methods.^56^ Given the nature of service provision in South Africa, and more generally throughout the continent, this will require much closer working between government, civil society, researchers and private sector organisations.^57,58^ While this paper has focused on the need to implement the CRPD health and rehabilitation articles, these are in reality embedded within the broader development agenda that countries embrace and so advocacy and targeted influencing of decision-makers are necessary at this level too.^59^

More generally there is a need to ensure not only that vulnerable and marginalized groups are equitably represented in policy documents, but that the process of policy development and its implementation, are undertaken in inclusive and empowering ways.^60^ Government programs to address disability must also take into account issues such as poverty which blight communities of persons with disabilities. Furthermore, since attitudes underpin most of the barriers, a comprehensive disability advocacy strategy that targets social institutions should be a fundamental element of any implementation plan. For positive practices to be implemented will require a change in attitudes from a range of different stakeholders. Human rights are not meant to be simply words on paper or principles to be held in the abstract. Moving them from policy into practice requires thinking about clear implementation pathways that cultivate a culture and builds a system that allows rights of real individuals to be realized on the ground.

## Ethical issues

The study received ethical approval from both Trinity College Dublin and the Health Research Ethics Committee, Dublin 2, Ireland (Application 06F/205/02) and the Stellenbosch University Faculty of Health Sciences, Cape Town, South Africa (Application S15/02/27).

Persons with disabilities can, in some circumstances, be considered a vulnerable group. This is particularly the case when a person has an intellectual disability that could render them unable to give informed consent.^61^ In certain South African contexts, it could also be the case that persons with disabilities in some areas experience high degrees of social marginalization, thus, making it appropriate to consider them a vulnerable group. For this ethical reason, the inclusion criteria for this study included being a representative of an organization that works with or on behalf of persons with disabilities, as these individuals have put themselves in a position as an advocate for this community. The persons with disabilities included in the sample were in such positions of organizational leadership and public advocacy. None had reported having intellectual disabilities, or impaired cognitive ability to consent.

All interviews included written informed consent. It was stressed that participation in the study was completely voluntary. Participants were always given the freedom to decline to answer a question or to stop the interview.

## Competing interests

Authors declare that they have no competing interests.

## Authors’ contributions

MH conceived of the study, acquired the data, performed the data analysis, and drafted the manuscript. Both MM participated in the design of the study and supervised the analysis of the data. GM supervised the collection of data in the research setting and aided with participant recruitment. Both MM and GM provided important critical revisions of the manuscript for intellectual content. All authors have read and approve of the final manuscript.

## Authors’ affiliations

^1^Centre for Global Health, Trinity College Dublin, Dublin 2, Ireland. ^2^School of Psychology, Trinity College Dublin, Dublin 2, Ireland. ^3^Centre for Rehabilitation Studies, Department of Interdisciplinary Health Sciences, School of Medicine and Health Sciences, Stellenbosch University, Cape Town, South Africa.

## Key messages

**Implications for policy makers**

- In the wake of the signing and ratification of the Convention on the Rights of Persons with Disabilities (CRPD), identification of the barriers to its full implementation are important to understand where gaps exist between policy and practice.
- Further research on strategies to overcome these barriers in the policy implementation process would benefit from active participation from members of the disability community.
- Greater sensitization towards disability needs to take place within the workplace and training of health personnel.
- More formal and informal linkages need to be established between different government departments and between government and civil society to increase accountability and coordination of services
- Audits of accessibility of health facilities and health information are needed to ensure equal access to healthcare for persons with disabilities.

**Implications for public**

This study’s findings show that a number of barriers: attitude, political, financial, health system, physical and communication, exist to the implementation of the rights of persons with disabilities regarding healthcare and rehabilitation services in South Africa. The findings from this study show a greater need for advocacy and involvement of the disability community to devise strategies to overcome these barriers.
